# Plant signals differentially affect rhizosphere nematode populations

**DOI:** 10.1093/jxb/erab149

**Published:** 2021-05-04

**Authors:** Ulrike Mathesius, Sofia R Costa

**Affiliations:** 1 Division of Plant Sciences, Research School of Biology, Australian National University, Canberra ACT 2601, Australia; 2 CBMA – Centre of Molecular and Environmental Biology, Department of Biology, University of Minho, Campus de Gualtar, 4710-057 Braga, Portugal

**Keywords:** Benzoxazinoids, nematode diversity, plant parasitic nematodes, rhizosphere signalling, root

## Abstract

This article comments on:

**Sikder MM, Vestergård M, Kyndt T, Fomsgaard IS, Kudjordjie EN, Nicolaisen M.** 2021. Benzoxazinoids selectively affect maize root-associated nematode taxa. Journal of Experimental Botany **72,**3835–3845.

**Plant health is significantly affected by the surrounding microbiome. While previous studies have progressed our understanding of how plants shape bacterial and fungal taxa in the rhizosphere, very little is known about plant signals shaping the nematode community in the rhizosphere. Using 18S rRNA metabarcoding and a secondary metabolite mutant defective in benzoxazinoid biosynthesis, Sikder *et al.* (2021) showed that plants can produce signals that selectively enrich or deter specific nematode taxa in and around their roots. This points to an active plant strategy to influence rhizosphere nematode populations through chemical signalling.**

In recent years, our knowledge on how plants actively manipulate their microbiome has increased rapidly. Multiple studies have demonstrated that root exudates can shape the microbial composition of the rhizosphere, with feedback effects on plant performance (e.g. Hartmann *et al.*, 2009). Most of these studies have focused on bacteria and fungi, but we know almost nothing about how the plant orchestrates the nematode microbiome.

**Box 1. What are benzoxazinoids and what do they do in the rhizosphere?**

Benzoxazinoids (BXs) are secondary metabolites derived from indole that are produced mainly by members of the Poaceae family, including wheat, rye, and maize (Zhou *et al.*, 2018). The functions of BXs, as well as some of their breakdown products, have been characterized during plant defence, primarily to insect herbivores and fungal pathogens. They act, for example, by inhibiting the digestive enzymes of insects. They have also been implicated in attracting beneficial rhizosphere bacteria, such as *Pseudomonas putida*, which chemotactically responds to certain BXs. In the plant, BXs are produced as active aglycones, but are stored as inactive glycosides in the vacuole, from where they can be released as aglycones following deglycosylation upon mechanical attack. Further breakdown can then occur due to the activity of soil organisms (Fig. 1). Both BXs and their breakdown products can be active. Their release into the soil has previously been shown to alter the composition of bacterial and fungal communities around maize roots (Cotton *et al.*, 2019; Kudjordjie *et al.*, 2019), and this has been linked to increased plant defences against plant herbivores (Hu *et al.*, 2018). Sikder and colleagues (2021) show here that BXs also shape nematode communities. This could be caused by specific attraction or repulsion of particular nematode taxa by BX metabolites, due to nematocidal activity, alteration of feeding behaviours of BXs, or more complex indirect interactions between BXs, nematodes, and other microbial taxa in the rhizosphere. The fact that BXs mainly affected plant parasites, namely root lesion nematodes, rather than free-living nematodes, could suggest possible involvement of chemotaxis or direct plant defence, or alternatively their higher exposure to BXs as they are located closer to the root than free-living nematodes; however, this will need to be tested.

An interesting additional complexity is that BXs have been shown to act across several trophic levels: BXs produced by plants in defence against insect herbivores can accumulate initially in the insect, which can become tolerant to the toxicity. Entomopathogenic nematodes, which infect insects, then ingest the BXs from the insect host, but they, too, have been shown, over time, to evolve tolerance to the BXs taken up from the insects (Zhang *et al.*, 2019). Further, entomopathogenic nematodes can harbour bacterial symbionts that may play a role in their detrimental effect on the infected insect host. It has recently been shown that those bacterial symbionts can also evolve tolerance to BXs contained in their nematode host (Machado *et al.*, 2020). These findings suggest that it is difficult to predict the exact mechanism of action of BXs or their breakdown products below- and above-ground, as these involve complex interactions between several kinds of organisms.

Nematodes are thought to be the most abundant multicellular organisms on Earth. They occur ubiquitously in soil and are distributed in all levels of the soil food web, intervening in numerous soil processes (van den Hoogen *et al.*, 2019). While most are seen as beneficial organisms, some have evolved very successful strategies to feed on plants. Plant parasitic nematodes are some of the most destructive pests, with world-wide estimated crop losses reaching 10–25% across a large number of crops, vegetables, turf gasses, and ornamental plants. Endoparasitic nematodes, which infect and reproduce inside host plants, cause physical damage to roots or form feeding sites that redirect nutrients from the plants for parasite feeding and reproduction (Jones *et al*., 2013). While some nematodes are fairly specific in their host range, many others are able to infect the majority of vascular plants. The reason for this is not well understood.

One strategy to defend against parasites and pathogens is to deploy chemical defences. Plants are very successful chemists, producing tens of thousands of metabolites that can be adapted as signals, toxins, and food sources potentially directed at microbes (Desmedt *et al.*, 2020). Depending on their water solubility or volatility, root-exuded metabolites may act locally in the rhizosphere or travel longer distances in the gaseous fraction of soil pores, respectively (Reynolds *et al.*, 2011). Some metabolites that may be ‘directed’ at one organism might also affect other organisms because they are publicly broadcast in the soil environment and, eventually, some of the soil biota evolve mechanisms to respond to these signals for their own benefit.

Parasitic nematodes can locate host roots by following non-specific cues of root biological activity, such as gradients of CO_2_ or pH, and plant-emitted volatile organic compounds, acting as long-distance nematode attractants. This may well be a generalist suite of molecules that attract nematodes, irrespective of whether they are plant parasites or free-living nematodes, to the rhizosphere. Locally, plant parasitic nematodes are attracted to root entry sites by compounds in highly diverse mixtures of metabolites present in host root exudates (Čepulytė *et al.*, 2018), including more complex molecules such as alkaloids, terpenoids, flavonoids, thiazoles, and benzoxazinoids (BXs) (Sikder and Vestergård, 2020). Other behavioural responses to plant secondary metabolites have been characterized, which can act in plant defence, inhibiting egg hatching, or being nematocidal (Sikder and Vestergård, 2020). Some of these metabolites also act on other organisms; for example, certain flavonoids can act as repellents to nematodes, at the same time as controlling symbiotic behaviour of rhizobia and acting on fungal hyphal growth (Hassan and Mathesius, 2012). Similarly, BXs play multiple roles in interkingdom signalling and defence, acting on insects, fungi, bacteria, and nematodes (see Box 1). The study by Sikder *et al.* (2021) clearly shows that individual BX metabolites are correlated with abundance patterns of particular nematode taxa, suggesting that they could act as signals. However, their mode of action on nematodes in the soil is still unclear. It will be fascinating to investigate whether production of BXs by the plant is targeted at nematodes in a specific way (i.e. responds to their presence), which behaviours are targeted, and if specific BX production confers a fitness advantage.

## Measuring soil nematode abundance and diversity

Nematologists have historically focused on plant parasitic nematodes in a plant protection perspective, with free-living nematodes frequently being discarded or omitted altogether from plant and soil studies. However, their roles in, for example, soil nutrient cycling through selective feeding on bacteria and fungi, and pest and disease suppression mainly through the action of higher trophic groups (omnivores and predators) are now widely recognized. In recent decades, foundations have been established for the assessment, analysis, and interpretation of soil nematode communities, including plant parasites and free-living nematodes, for use as bioindicators of soil health status and functions (Ferris *et al.*, 2001; Ferris, 2010). A biogeographical study of soil nematode communities at the global level, including >6750 samples representing all biomes and continents, revealed large patterns and environmental drivers of nematode abundance and functional diversity. As with other animal groups, nematode abundance is affected by climate (temperature and precipitation), but it is mainly driven by soil physical–chemical properties, such as texture, pH, and organic matter content. The latter affects nematodes in all trophic groups similarly and is thought to ultimately determine the size of the soil food web. Global patterns in densities of plant parasitic nematodes respond to vegetation cover and photosynthetic activity, whereas free-living bacterivores are more affected by soil properties (van den Hoogen *et al.*, 2019, 2020). Such large-scale spatial modelling efforts cannot be informative on drivers of nematode abundance and diversity at local scales. However, local assessments of soil nematode communities have been conducted in a plethora of field and controlled conditions, to investigate, for example, effects of climate and land use change, soil contamination by various agents, impacts of agricultural practices, and plant–soil feedbacks.

Classical assessments of soil nematode communities typically consider morphological identification of 100–200 nematodes, extrapolating to total live, active nematodes extracted from a 100 g soil sample, that frequently pools bulk soil, small root fragments, and adhering rhizosphere soil. Nematodes are classified in trophic groups, or to at least family level to be classified in functional guilds (Bongers, 1990; Yeates *et al.*, 1993). Due to morphological identification limitations and the broader aims of functional ecology analyses, classical assessments usually consider identification only up to genus level.

Molecular tools have been providing rapid, high-throughput identification and quantification up to species level, thus expanding on nematode community analysis, and increasing our understanding of finer plant–soil interactions (e.g. Schenk *et al.*, 2019; Sikder *et al.*, 2021). Small sample sizes and uniform extraction methods allow for the meticulous quantification of several nematode taxa and their temporal variation in different compartments—bulk soil, rhizosphere, and roots—used in Sikder *et al.* (2021) to elucidate the differential effects of BXs on plant parasitic and several free-living nematode taxa. The (agro)ecological consequences of these findings can be exploited to assess effects of unweighted selective pressures on plant parasitic species, as well as the indirect or unintended effects on free-living nematodes, that can lead to changes in rhizosphere nutrient turnover (through effects on bacterivores and fungivores) and natural regulation of plant parasitic nematodes (through effects on omnivores and predators).

## Looking ahead

While many metabolites have been identified that have effects on soil nematodes *in vitro*, we know very little about their actual effects in the field—are their concentrations appropriate to affect behaviours; which organisms metabolize them and to which end products; and how are the metabolite activities affected by soil pH, moisture, soil type, and other organisms present?

Another major gap is the lack of understanding of how phytochemicals actually work in nematodes. What their molecular targets are and how they are perceived is largely unknown. While *in vitro* assays have differentiated the effect of many pure compounds on nematode egg hatching, movement, or chemotaxis, the target proteins/receptors remain to be discovered.

**Fig. 1.**
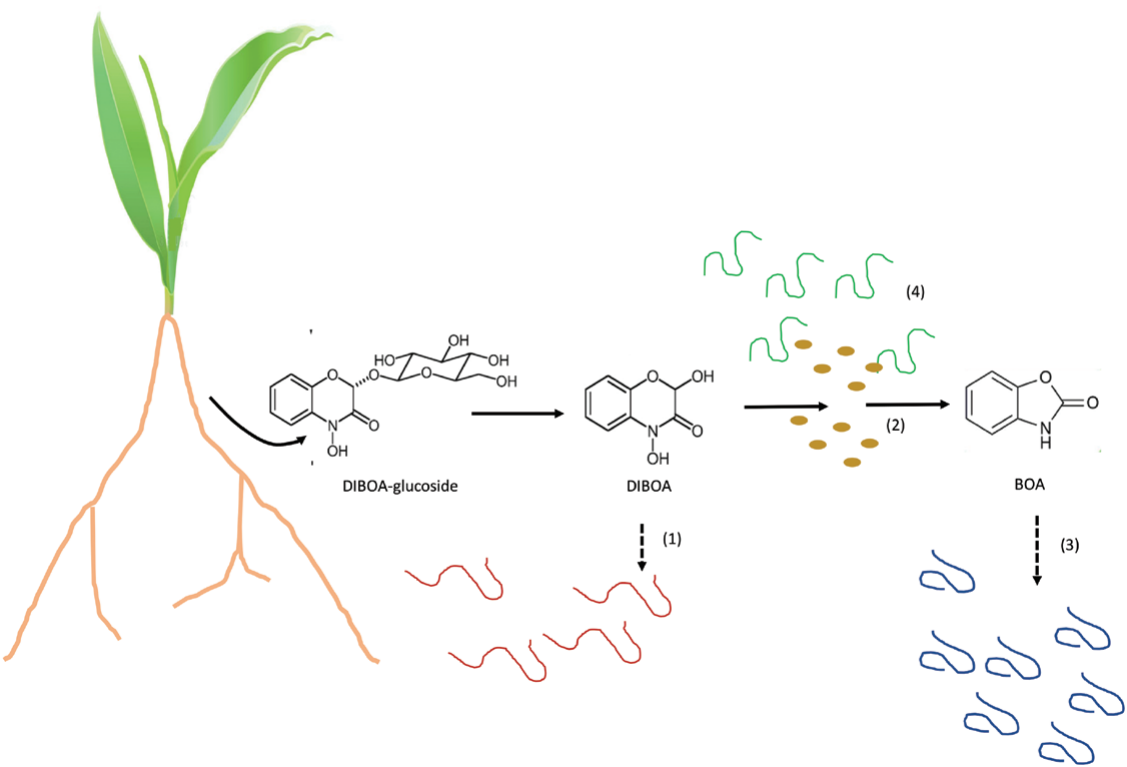
Interactions between roots, benzoxazinoids, and soil nematodes. The root stores benzoxazinoids as glycosides, for example DIBOA-glucoside (2,4-dihydroxy-1,4-benzoxazinone glucoside), which is deglycosylated into DIBOA. (1) DIBOA could have direct effects on attracting or repelling, for example, parasitic nematodes (red) to/from the root. (2) DIBOA can be broken down to BOA (benzoxazolin-2-one), for example by bacteria. (3) BOA could have a specific effect on different taxa of nematodes (blue). (4) Bacterial-feeding nematodes (green) could affect the bacterial populations that metabolize benzoxazinoids, indirectly altering the breakdown products and their targets. The figure is a simplified diagram to exemplify the concept. As shown by Sikder *et al.* (2021), multiple other BXs and breakdown products are involved.

It should also be kept in mind that studies on the influence of plant metabolites on the microbiome are difficult to predict in their entirety. For example, changes in root metabolites can have indirect effects on other metabolites that could be active as signals or cues; breakdown products of the metabolites in the soil can be unpredictable and can depend on the many species of soil organisms that can produce such breakdown products as well as physical and chemical soil conditions (Fig. 1). In addition, the study by Sikder and colleagues showed that effects of BXs changed over time. A possible explanation is that the molecules could have rapid effects on chemotaxis and slower effects via affecting egg hatching, which would only be detected after a lag time in the next generation. BX production is also developmentally regulated, so the temporal effect could partly reflect differences in BX production or release as the plant matures. Chemical signals are also likely to differ spatially in the soil as many metabolites are exuded from specific parts of the root system, such as the root tip or sites of lateral root emergence. The rhizosphere remains one of the most complex ecosystems on our planet!

How could the information on the role of plant chemical signals on nematode soil ecology be exploited in agriculture (Hiltpold and Turlings, 2012)? Possible approaches include breeding of crops with altered concentrations of specific chemicals, keeping in mind possible off-target effects on other organisms. It is also possible to produce chemical baits that could be dispersed into the soil in capsules to manipulate the behaviour of soil-dwelling nematodes; this would have to be specific enough to attract parasitic nematodes while avoiding removal of beneficial nematodes from the soil. Targeted crop rotation and intercropping with plants attracting particular nematode taxa away from the main crop are another option. With limited resistance and no effective and safe control method available for the control of parasitic nematodes, a phytochemical approach remains a sustainable option. The ability to screen for thousands of phytochemicals using untargeted metabolomics, coupled with more sensitive methods for detecting and quantifying a large range of soil microorganisms, provides many future opportunities in this area.
